# Resurgence of Cutaneous Leishmaniasis in Israel, 2001–2012

**DOI:** 10.3201/eid2010.140182

**Published:** 2014-10

**Authors:** Dan Gandacu, Yael Glazer, Emilia Anis, Isabella Karakis, Bruce Warshavsky, Paul Slater, Itamar Grotto

**Affiliations:** Israel Ministry of Health, Jerusalem, Israel (Y. Glazer, D. Gandacu, E. Anis, I. Karakis, B. Warshavsky, P. Slater, I. Grotto);; Hebrew University and Hadassah–Braun School of Public Health, Jerusalem (E. Anis);; Ben-Gurion University of the Negev Department of Public Health, Be’er Sheva, Israel (Y. Glazer, I. Karakis, I. Grotto)

**Keywords:** cutaneous leishmaniasis, zoonosis, epidemiology, resurgence, Israel, Leishmania, parasites

## Abstract

Cutaneous leishmaniasis has long been endemic in Israel. After a 15-year period of moderate illness rates, reported incidence increased from 0.4 cases per 100,000 population in 2001 to 4.4 cases per 100,000 population in 2012, and the disease emerged in areas where its presence had previously been minimal. We analyzed all cases reported to the national surveillance system and found that outbreak patterns revealed an expansion of *Leishmania major* infections over large areas in the southern part of the country and the occurrence of spatially focused *L. tropica* outbreaks in the northern part of the country. Outbreaks often followed new construction in populated areas. Further study of factors affecting the transmission of cutaneous leishmaniasis is needed in Israel, as well as the development of effective methods to control the disease, an increase in awareness among health care professionals, and intensive public education regarding control measures in areas of known leishmaniasis foci.

Leishmaniasis is a disease caused by parasites of the genus *Leishmania *(Trypanosomatidae: Kinetplastida); global incidence approaches 2 million cases annually (*1**,**2*). Cutaneous leishmaniasis (CL), the most common form of the disease (*3*), is endemic in most Mediterranean countries (*4*). Humans become hosts of the disease when the parasitic infection develops in the immune system and causes skin lesions. The lesions tend to heal spontaneously after 3–18 months (*5*) but often result in disfiguring scars (*6**,**7*). Functional complications are rare (*8*).

CL has long been endemic in Israel, and the disease is known colloquially in the region as the “Rose of Jericho.” Historically, the main source of the disease in Israel has been *L. major* parasites; cases resulting from this species have been widely distributed in the Negev region in the Southern health district, the arid and semi-arid area of southern Israel that that is sparsely populated and accounts for ≈60% of the country’s land. More recently, illness caused by *L. tropica* parasites has been reported in several semi-arid hilly areas in Israel’s more densely populated, and less dispersed, central and northern population centers (*9**,**10*).

The recognized vector of CL in Israel is the female phlebotomine sand fly. The species predominantly responsible for CL cases in Israel have been *Phlebotomus papatasi* for *L. major* infections and *Ph. sergenti* for *L. tropica* infections. The reservoir of *L. major* parasites consists of rodents (e.g., *Psamomys obeesus, Meriones crassus*, *Microtus guentheri*, *Meriones tristrami*, *Gerbillus* spp.), whereas the main reservoir of *L. tropica* parasites is the rock hyrax (*Procavia capensis*). *L. tropica* infection tends regionally to be an urban, anthroponotic phenomenon, but in Israel it is zoonotic in nature and has an incubation period that is longer than that for *L. major* infection and is more resistant to treatment. *L. tropica* infection also results in multiple lesions and, when it results in leishmaniasis recidivans, has a lower tendency to heal spontaneously (*11**,**12*).

Before 2001, a total of 3,352 cases of CL had been reported in Israel, and annual incidence ranged from 0.1 to 7.3/100,000 population. Two periods of particularly high illness rates occurred during this time, in 1967–1969 and in 1980–1982 (*13*). The first peak, in the late 1960s, appeared after the June 1967 War, after the exposure of naive populations to the parasite in disease-endemic areas. The second peak, in the early 1980s, was assumed to have resulted primarily from the continuing increase in the number of new settlements westward from the Jordan Valley toward Jerusalem (*13*). A decade and a half of relatively moderate incidence followed, and at the end of 2000, reported national incidence stood at 0.3/100,000 population. However, by 2012, reported incidence had increased to 4.4/100,000 population and CL had emerged in areas where its presence had previously been minimal. We describe these changes from an epidemiologic point of view and discuss factors that might explain the increase in CL rates and distribution from 2001 to 2012.

## Methods

The Israeli Ministry of Health has conducted routine national surveillance of CL since 1949, when the disease became reportable. Cases are reported through a computerized notification system network from each of 15 regional health districts in the country. The ministry’s Division of Epidemiology (DOE) monitors and processes the case report data on a national basis. Although the surveillance method is passive and is assumed to underestimate the actual number of CL cases, no changes in reporting methods and no official efforts to increase the frequency of notification have been implemented. Therefore, we assumed in our research that the reported incidence data are a good indication of actual epidemiologic trends in the population.

Two caveats should be noted regarding any CL incidence data. Underreporting of CL is widely acknowledged to be a problem for a variety of clinical, logistic, cultural, and other reasons (*14*); we discuss some of the underlying causes for underreporting later in this article. However, during major outbreaks or when infection occurs in previously non–CL-endemic areas, heightened awareness may increase the proportion of CL cases that are officially reported.

Laboratory confirmation of suspected CL cases is currently not required, and therefore, laboratory tests are not routinely performed on samples from all reported case-patients. Consequently, both probable and confirmed CL cases are reported to the DOE, and distinctions cannot be made in the national incidence data between *L. major* and *L. tropica* infections. Because the laboratory results that are available provide an indication of the *Leishmania* species, these results can be used to make reasonable species-related assumptions regarding specific outbreaks and regarding other reported cases in nearby geographic areas where another species has not been detected. However, for those areas in which >1 species of *Leishmania* is found, such assumptions cannot be made (*15*).

We analyzed all CL cases that were reported to the Israel national surveillance system for which onset occurred during January 1, 2001–December 31, 2012. For methodologic reasons, all cases reported here are from the civilian population; the Israeli military uses active surveillance for CL and commonly includes only cases with a clinical diagnosis. 

The denominators used for calculations of crude and specific incidence rates per 100,000 persons according to age, sex, population group, and health district were derived from annual population estimates from the Central Bureau of Statistics of Israel (*16*). We used χ^2^ tests for comparison between rates. All statistical analyses were conducted by using Microsoft Excel 2010 (Microsoft, Redmond, WA, USA) and SPSS version 19.0 (SPSS IBM, Armonk, NY, USA). We considered p<0.05 as statistically significant.

## Results

Overall, 2,061 cases of CL that occurred in Israel were reported during January 1, 2001–December 31, 2012. Of the case-patients, 58% were men, and 93.8% were Jewish. The mean age at disease onset was 31.1 + 22.1 (range 0–90, median 28) years. More than 42% of reported cases occurred during 2010–2012, and 40% of case-patients were residents of the Southern health district/Negev region.

**Figure 1:**
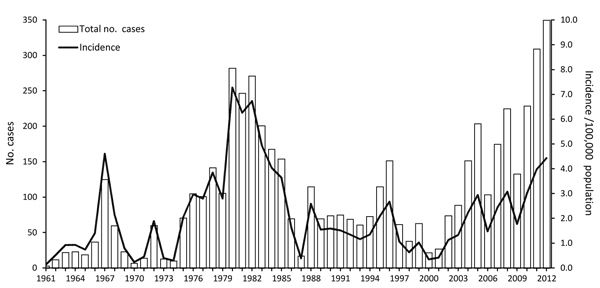
Cutaneous leishmaniasis in Israel, 1961–2012, showing annual number of cases and incidence per 100,000 population. A sharp increase is shown for the study period, 2001–2012.

**Figure 2:**
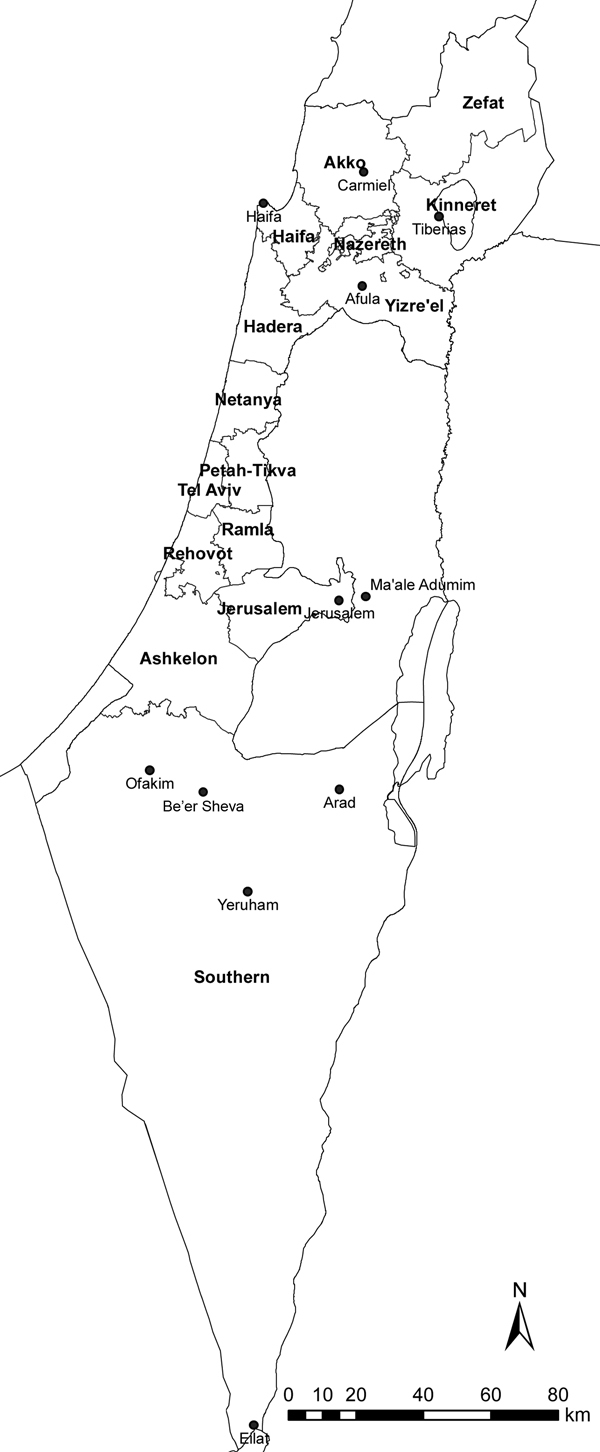
Selected localities (black dots) where cases of cutaneous leishmaniasis were reported in Israel during 2001–2012. Health districts are labeled in boldface.

From 2001 to 2012, the CL incidence rate in Israel increased tenfold, from 0.4 to 4.4/100,000 population (Figure 1). Over this period, mean annual incidence increased by 0.36/100,000 population. These increases reflect, in part, the spread of CL to central and northern areas of the country, where reports of the disease had previously been minimal. Those increases were highlighted by several outbreaks and incidence peaks. An outbreak occurred in the Kinneret subdistrict near Lake Tiberias in northern Israel in 2003; several dozen cases were reported, sometimes >1 per family, in 2 new periphery neighborhoods at the edge of the city of Tiberias. During 2004–2005, an outbreak of >100 cases occurred in 2 new neighborhoods near the ravines of a suburb, Ma’ale Adumim, in the Jerusalem health district. A peak in national incidence occurred in 2007–2008, a result of an increase in CL cases in the Southern health district, where *L. major* parasites are endemic, and an outbreak of *L. tropica* infections in the northern district. Whereas cases from the southern region were sporadically distributed, in the northern region, cases were restricted to peripheral houses near the borders of 1 rural settlement in the Yizre’el subdistrict, where few cases had been reported in the past. The main sources of a subsequent peak in 2012 were the occurrence of 37 cases in Ofakim, a small town in the Southern health district, and 44 cases that occurred with the continuation of the outbreak in Ma’ale Adumim (24.2% and 64.7% of the district totals, respectively). Toward the end of 2012, a new outbreak began in Carmiel, in the Akko subdistrict. CL illness rates also increased within the Southern health district in several localities, including Be’er Sheva, Eilat, Yeruham, and Arad, and expanded elsewhere to other, much smaller, rural villages (Figure 2).

Among age groups, increases in incidence were highest during most years among infants. Nevertheless, the 15–44-year-old age group comprised the greatest portion of case-patients each year compared with the other age groups; these differences were significant in 2006 (p = 0.03), 2007 (p = 0.016), 2009 (p = 0.025), 2010 (p<0.001), and 2012 (p<0.001) (Table). CL incidence was generally higher among the male population than among the female population, except for those >65 years of age.

**Table:** Annual cutaneous leishmaniasis incidence and proportion of total cases, by age group, Israel, 2001–2012

Year	Incidence/100,000 population, by age group, y						Proportion of total cases, by age group, y				
	<1	1–14	15–44	45–64	≥65		<1	1–14	15–44	45–64	≥65
2001	0.0	0.6	0.4	0.3	0.3		0.00	0.37	0.41	0.15	0.07
2002	2.2	1.3	0.9	1.4	1.1		0.04	0.30	0.34	0.23	0.09
2003	2.1	1.4	1.3	1.6	0.6		0.03	0.28	0.42	0.22	0.04
2004	4.9	2.0	2.6	1.9	1.0		0.05	0.24	0.51	0.16	0.05
2005	2.1	3.5	2.3	3.2	3.9		0.01	0.31	0.34	0.20	0.13
2006	4.1	1.9	1.2	1.4	1.0		0.06	0.35	0.36	0.17	0.07
2007	5.4	2.7	2.4	3.0	0.3		0.05	0.29	0.42	0.23	0.01
2008	3.9	3.7	2.8	3.6	1.5		0.03	0.32	0.39	0.22	0.05
2009	2.5	2.5	1.5	1.6	1.1		0.03	0.37	0.37	0.17	0.06
2010	4.9	3.1	2.3	4.5	2.5		0.03	0.27	0.32	0.29	0.08
2011	6.7	3.5	3.7	5.0	3.4		0.04	0.23	0.39	0.24	0.09
2012	1.8	4.0	3.8	5.8	4.7		0.01	0.24	0.37	0.26	0.11

**Figure 3:**
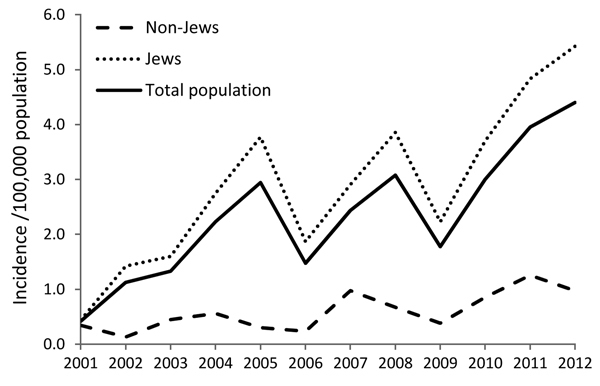
Annual incidence of cutaneous leishmaniasis by population group (Jews vs. non-Jews), Israel, 2001–2012.

Reports of CL during 2001–2012 were markedly dissimilar by population group. More than 93.8% of reported case-patients were Jewish (range 81.5%–94.4%), even though Jews made up only 75%–77% of the national population during the study period (*17*). Similarly, annual incidence rates for Jews were consistently higher than those for non-Jews (p = 0.032). The mean incidence rate for Jews was 2.9/100,000 (range 0.4–5.4) versus 0.6/100,000 (range 0.1–1.3) for non-Jews (Figure 3).

## Discussion

Trends in reported CL in Israel during 2001–2012 indicate an increase in the incidence of the disease, particularly in recent years, and the spread of CL to new areas. Regional patterns in reported incidence rates show numerous outbreaks of CL spreading beyond the Southern health district, which had traditionally been the source of most cases in Israel, to regions of the country in which reports of the disease had previously been minimal. Whereas CL in the south is mostly endemic and occurs sporadically over large areas (the sporadic nature of *L. major* infection has also been noted in nearby countries [*18*]), the outbreaks in the north have been more focused both temporally and spatially. In the 2003 outbreak in Tiberias, for example, CL cases appeared within a short time span and often infected family clusters. An *L. major* outbreak that occurred in the eastern area of the Yizre’el subdistrict (Beit She’an valley) was similarly focused and distinct from outbreaks in southern areas in Israel to which *L. major* parasites are endemic. In the latter outbreaks, reported cases typically had no epidemiologic linkage in time or place and occurred in such localities as Yeruham, Dimona, Ofakim, and various rural villages that are widely dispersed throughout the southern region of the country.

Many of the CL outbreaks in the northern region of Israel were notable for the emergence of *L. tropica* parasites, which had not previously been known to be a common cause of CL in Israel. The increasing incidence of *L. tropica* infections has also been observed in other countries in the Mediterranean Basin and elsewhere in Asia (*19**,**20**,**21*). In addition to the resulting increase in CL incidence, the presence of *L. tropica* parasites poses other concerns. Infections caused by this species tend to be more diffuse and difficult to treat than infections caused by *L. major* parasites and can sometimes lead to more chronic or more dangerous forms of the disease, such as leishmaniasis recidivans or, in rare cases, visceral leishmaniasis.

The incidence of CL has been increasing globally during the past decade (*22*). Research into the reasons for this occurrence has identified human activity and interventions in the environment (e.g., migration, urbanization, and deforestation [*23**–**26*]), longer-term or natural events (e.g., climate change [*27*] and earthquakes [*28*]), and improvements in the detection and diagnosis of the disease. In Israel, the increase in CL incidence rates several decades ago was explained by immigration of naive populations into endemic areas, but today, this reason is applicable only to persons from non–CL-endemic areas who visit CL-endemic regions. The most probable causes for increased incidence in more recent years are the changes to the vector-reservoir-human population network resulting from land development and construction, the expansion of populated areas, and changes in land use (such as in Tiberias, Ma’ale Adumim, and Ofakim). These factors have altered the relationships of human communities with the surrounding natural environment and have created favorable conditions for mammalian reservoirs and sand fly populations to breed in close proximity to human habitations (*23*,*24*), a phenomenon that has also occurred elsewhere (*29*). This combination of events has facilitated transmission of CL in established populated areas among persons who had lived there for many years, as well as the spread of the parasite to new locations. 

Climate change may also contribute to the spread of CL by influencing (e.g., through increased temperatures or rainfall) variables such as vector life cycles, mammalian reservoir abundance, disease transmission patterns, and the geographic scope of the pathogen (*30**,**31*). Preliminary research has shown a high correlation between temperature increases and the incidence of human CL in Israel (*32*). 

Examination of monthly reporting patterns over the past several decades showed a shift in the seasonal peak of CL case reports from the summer to the autumn. This change in seasonality may reflect the population dynamics of the sand fly vector for *L. tropica* parasites (*33**–**35*) compared with the vector for *L. major* parasites. Further study is needed to clarify whether the continuing emergence of *L. tropica* parasites in the northern region of Israel is partly responsible for the seasonality shift or whether other causative factors are involved.

Reported CL incidence in Israel has consistently been higher among the male population, except among persons >65 years of age, which correlates with findings from studies in other CL-endemic countries in which the relationship between sex, age, and CL has been addressed (*29*,*36*). Similarly, the total number of reported CL cases in Israel is highest among adults of working age, as has also been found elsewhere (*37*). However, in almost every year during the study period, the CL incidence rate for infants was greater than for any other group, which may reflect diagnoses made during routine well-baby care examinations that are provided at Israel’s Mother and Child clinics. If so, this finding may be a cause of information bias (diagnostic bias) in the case surveillance system, a reflection of the fact that infants do not have immunity to CL, as may be the case with children and adults. Because of data limitations in the reporting method used in this study, the age and sex attributes could not be tested for confounders or effect modifiers that could result from other factors, such as lifestyle, working place, time spent outdoors during sand fly activity hours, and insect repellent usage.

A notable difference continues to appear in the CL reporting rates among the country’s major population groups. The reported incidence rates for the non-Jewish population were fairly constant and were lower during the study period than those for the Jewish population. Because of the limitation of the passive surveillance reporting system, it is difficult to know exactly how much of this difference reflects actual incidence and how much results from other factors, such as the influx of non–CL-immune Jews into existing disease foci or the preference in some non-Jewish communities in CL-endemic areas not to seek treatment for a familiar skin lesion. The difference may also be explained in part by other cultural and behavioral traditions that can influence health-seeking behavior, particularly because the gap in incidence has remained constant over the years. In some CL-endemic areas in the Middle East and southern Asia, for example, the appearance of permanent scarring after CL infection may lead to social consequences such as shame, stigmatizing effects, or harm to a woman’s chances for marriage (*38*,*39*). Because CL infection provides immunity from future infection by the same *Leishmania* species, a common folk custom among some population groups is to proactively acquire infection with the parasite to generate natural immunity against future disease. This process involves deliberately exposing infants, especially girls, to the bite of a phlebotomine sand fly on a part of the body that is normally covered by clothing (*40*). This practice may continue to some extent in Israel among some desert-dwelling Bedouin; however, we believe the practice has become far less prevalent in Israel among this population subgroup because of continuing trends toward modernization and urbanization.

Assuming that the underreporting of actual illness was fairly constant during the years of study, the incidence data we present show cyclical trends that reasonably reflect the 2 main currents of human CL in Israel during the 2001–2012 period: 1) the sporadic CL that has long been endemic to southern Israel; and 2) the occurrence of focused CL outbreaks resulting from population movement, land development, and the emergence of *L. tropica* parasites in other areas of the country*.* Although passive surveillance does not permit a more accurate estimation of the full extent of CL disease in Israel, the reported incidence data clearly show an expansion of CL to areas in which CL was not previously known to be endemic. The concern that reported CL cases not only have been increasing in volume but also are emerging in geographic areas where illnesses previously had been rare has led an inter-ministerial committee to institute a National Program to Reduce Leishmaniasis Hazards in Israel. This 3-year program, led by the Ministry of Environmental Protection in collaboration with the Ministries of Health, Defense, and Interior, and the Nature and Parks Authority, includes the experimental implementation of different intervention methods designed to reduce disease transmission and increase personal protection in 50 localities, as well as the initiation of scientific and feasibility studies of relevant zoonotic, epidemiologic, and control issues.

In summary, a review of nationwide outbreaks over a recent 13-year period has revealed an expansion of CL illness in southern, central, and northern areas of Israel. Regional differences were seen regarding the dominant causative species for infection and the geographic and temporal patterns of outbreaks. Further study of the factors affecting CL transmission is needed, as are the following: development of effective methods to control CL infection, an increase in health professionals’ awareness of the disease, and a greater degree of public alertness. Intensive public education regarding control measures in areas of known leishmaniasis foci is required.
